# Disruptions in Oncology Care Confronted by Patients with Gynecologic Cancer Following Hurricanes Irma and Maria in Puerto Rico

**DOI:** 10.1177/10732748221114691

**Published:** 2022-07-14

**Authors:** William A. Calo, Mirza Rivera, Pablo A. Mendez-Lazaro, Sandra I. Garcia-Camacho, Yanina M. Bernhardt Utz, Edna Acosta-Perez, Ana P. Ortiz

**Affiliations:** 1 Department of Public Health Sciences, 12310 Penn State College of Medicine, Hershey, PA, USA; 2 12310 Penn State Cancer Institute, Hershey, PA, USA; 3 Center for Evaluation and Sociomedical Research, Graduate School of Public Health, 12320 University of Puerto Rico Medical Sciences Campus, San Juan, PR, USA; 4 Department of Environmental Health, Graduate School of Public Health, 12320 University of Puerto Rico Medical Sciences Campus, San Juan, PR, USA; 5 Division of Cancer Control and Population Sciences, 591857 University of Puerto Rico Comprehensive Cancer Center, San Juan, PR, USA; 6 Hispanic Alliance of Clinical and Translational Research, 12320 University of Puerto Rico Medical Sciences Campus, San Juan, PR, USA; 7 Department of Epidemiology and Biostatistics, Graduate School of Public Health, 12320 University of Puerto Rico Medical Sciences Campus, San Juan, PR, USA

**Keywords:** Puerto Rico, US Virgin Islands, gynecologic cancer, Oncology care, hurricanes, disaster response

## Abstract

**Background:**

In September 2017, hurricanes Irma and Maria affected Puerto Rico (PR) and the US Virgin Islands (USVI), causing major disruptions in basic services and health care. This study documented the stressors and experiences of patients with gynecologic cancer receiving oncology care in PR following these hurricanes.

**Methods:**

We conducted 4 focus groups (December 2018-April 2019) among women aged ≥21 years from PR who were diagnosed with gynecological cancer between September 2016 and September 2018 (n = 24). Using the same eligibility criteria, we also interviewed patients from the USVI (n = 2) who were treated in PR. We also conducted key-informant interviews with oncology care providers and administrators (n = 23) serving gynecologic cancer patients in PR. Discussions were audio-recorded, transcribed verbatim, and coded to identify emergent themes using a constant comparison method.

**Results:**

Analyses of focus group discussions and interviews allowed us to identify the following emergent themes: 1) disruptions in oncology care were common; 2) communication between oncology providers and patients was challenging before and after the hurricanes hit; 3) patient resilience was key to resume care; and 4) local communities provided much-needed social support and resources.

**Conclusions:**

This study provides firsthand information about the disruptions in oncology care experienced by and the resiliency of women with gynecologic cancer following hurricanes Irma and Maria. Our findings underscore the need to incorporate oncology care in the preparedness and response plans of communities, health systems, and government agencies to maintain adequate care for cancer patients during and after disasters such as hurricanes.

## Introduction

On September 6, 2017, Hurricane Irma hit the northeast region of Puerto Rico (PR) as a category 5 storm, leaving over 1 million residents without electricity.^1^ Two weeks later, Hurricane Maria made landfall. The category 4 hurricane hammered PR with sustained winds of 155 miles/hour for almost 30 hours and left areas with as much as 35 inches of rain and 100% of PR without electricity.^2,3^ Hurricane Maria became the third costliest storm in U.S. history, seriously affecting or completely destroying 472 000 homes.^3^ For weeks, or even months in some areas, potable water was scarce, or under boiling alert, telephone communications were lost, roads were blocked with debris or landslides, and many families were isolated because of collapsed bridges.^4^ Thousands of people were evacuated from their homes to shelters elsewhere on the island, and ≥200 000 Puerto Ricans were relocated to the continental US.^5^ Studies estimate the excess mortality from Hurricane Maria in PR between 1085 and 4645 deaths.^6,7^

The health care system in PR was also severely impacted.^4,8-11^ Only a handful of the 69 hospitals on the island were operational within the first weeks after Hurricane Maria made landfall.^8^ The medical facilities that did not suffer substantial infrastructure damage continued offering limited services with power generators.^8-10^ In addition, medical staff, patients, and suppliers had difficulty getting to the hospitals because of the severely damaged roads on the island. Since most communication systems were not functioning, neither hospitals, medical staff, nor patients could communicate with one another.^4,8^ Many smaller clinics, pharmacies, and laboratories were down for months or never opened again.^8^ Several recent reports have described the island-wide disruptions these hurricanes had on dialysis services,^10^ organ donation and transplants,^12^ immunizations,^13^ and delivery of prescription medications.^14^ Both hurricanes also caused major damages to the health care system in the neighboring territory of the U.S. Virgin Islands (USVI)^15^; whose population also receives care in PR.

Patients undergoing cancer treatment are among the most vulnerable populations following a hurricane, given the disruptions in care delivery.^16-18^ In PR, cancer is the leading cause of death, and gynecologic cancers represent more than 15% of all new cancers diagnosed.^19^ A study from the American Society for Radiation Oncology (ASTRO) found that 7 of 18 radiation oncology clinics in PR were reachable after hurricane Maria and they were only treating between 20% and 50% of their patients.^20^ Those numbers are alarming because patients with gynecologic cancer receiving radiotherapy have a 10% chance of treatment failure if they miss 2 or more treatments during a 4-week radiotherapy course.^21^

As hurricanes are increasing in both frequency and magnitude,^22^ it is critical to better understand the impact of hurricanes on cancer care. Such information would help health care administrators, oncologists, and patients to prepare and adopt plans to mitigate the potential impact of future storms on oncology care. This is especially important for gynecologic cancer as it represents a large number of female cancers in PR and the USVI. The objective of the present study was to document the stressors and experiences of patients with gynecologic cancer regarding their oncology care in the aftermath of hurricanes Irma and Maria. To meet our objective and have a broad perspective on the issue, we interviewed gynecologic cancer patients and oncology care providers and administrators.

## Methods

### Recruitment and Data Collection: Gynecologic Cancer Patients

In 2018, we partnered with 4 oncology clinics from various regions in PR to identify potential study participants. Eligibility criteria included women aged ≥21 years from PR or the USVI who were diagnosed between September 2016 to September 2018 with any of the following gynecologic cancers, based on the International Classification of Diseases for Oncology, third edition (ICD-O-3) codes: vulva (C51), vagina (C52), cervix uteri (C53), corpus uteri (C54), uterus, NOS (C55), ovary (C56), or other unspecified female genital organs (C57). Collaborating physicians from these clinics presented the study to 34 women, of which 3 were not interested. Then, the clinics provided the contact information of the 31 eligible women to the study coordinator, who then contacted them by phone to invite them to the focus groups. Among these women, 24 (response rate: 77%) agreed to participate.

We conducted 4 focus groups from December 2018 through April 2019. Two groups were conducted in San Juan, the capital city of PR (the northeast region of the island); one with publicly insured patients (n = 6) and another with those privately insured (n = 6). The other 2 groups were conducted with a mix of privately and publicly insured patients in Mayaguez (west region; n = 7) and Ponce (south region; n = 5). Group discussions were held at the 4 partnering oncology clinics as these locations were accessible to and well known by the study participants. Two patients from the USVI, who received oncology care in PR, were also interviewed individually through the phone given that they were in the USVI at the time of this study.

The study team developed the interview guide from previous instruments used to explore overall health care disruptions following natural disasters, including hurricanes,^16,23^ and from the own experience of study team members who experienced hurricanes Irma and Maria firsthand. The present study focuses on questions about cancer care, communications, family support, and social networks (Table 1). Prior to starting the focus groups or interviews, the study coordinator described the study and obtained informed consent. Cancer patients from Puerto Rico provided written consent and the 2 patients from USVI provided verbal consent over the phone. All participants consented to take part in the interviews by answering our questions, that the discussions be audio-recorded, and for the findings resulting from the interviews to be used for research purposes. Two facilitators led the discussions (PML, who has experience leading post-disaster focus groups, and MR, who has experience leading qualitative studies with cancer patients) while the study coordinator took notes. Focus groups were conducted in Spanish, ranged from 90 to 120 minutes in length, and were audio-recorded. The 2 interviews with patients from USVI were conducted in English, averaged 60 minutes in length, and were audio recorded. Participants received $30 as compensation for their time.

**Table 1.** Interview Questions from Focus Groups and Interviews with Gynecologic Cancer Patients.

Topic	Interview Guide Questions
Cancer care	1. After the hurricane, how was access to your cancer treatment affected? Did you have to seek medical care in other hospitals or clinics (in PR or outside of PR) since your doctor was not available? If so, was the treatment the same quality? 2. After the hurricane, did you receive information or help from your municipality, from FEMA, from the central or other government, to manage your health care? If so, what agency guided or helped you and how was your experience? 3. After the hurricane, was the treatment available at the time it was your turn to receive it? If it was not, were you notified that it was not available? How did they notify you? What reasons were given for it not to be available? How long was your treatment not available? And did they provide you with any solution to meet the need for your treatment? 4. After the hurricane, how was your cancer condition affected? 5. After the hurricane, how was your health treatment affected in your home? 6. After the hurricane, did you develop any condition or disease because your medical condition at that time was aggravated by the lack of necessary medical services? 7. Did you develop any condition or disease related to the hurricane?
Communication	8. After the hurricane, how did the clinics or organizations that give you cancer treatment keep in touch with you? 9. How did you stay informed of what was happening during the hurricane? 10. What was the source of communication that offered the most complete information, after the hurricane?
Family support	11. After the hurricane, how were relationships in your family affected? If your condition worsened, did any family member have to take care of you in terms of your health care? 12. If you have dependent minors, how were they affected in family relationships?
Social networks	13. After the hurricane, what kind of support in terms of helping your health, accommodation, money, food, among others, did you receive from your friends? 14. After the hurricane, how was the relationship between your friends affected?

### Recruitment and Data Collection: Oncology Care Providers and Administrators

We conducted key-informant interviews from December 2018 through April 2019. Participants were a range of oncology care providers and administrators (n = 23) who provide services to women with gynecologic cancers in PR, including oncologists, radiotherapists, pharmacists, hospital administrators, and representatives from government agencies and nonprofit organizations (eg, Department of Health, Office of the Patient Advocate, Cancer Control Coalition, the American Cancer Society). Similar to interviews with patients, the study team developed a semi-structured interview guide based on the literature^16,23^ and the researchers’ awareness of the needs and experiences of providers and organizations serving gynecologic cancer patients. Of note, the study team held quarterly meetings with a community-clinical advisory board to inform this project. Prior to starting the interviews, the study coordinator described the study and obtained informed consent from participants. Interviews took place in person or over the phone, ranged from 30 to 45 minutes in length, and were audio-recorded. The present study focuses on questions regarding problems encountered in their health care settings or organizations in the aftermath of hurricanes Irma and Maria, including damaged infrastructure, lack of electricity and potable water, issues with electronic medical record or communication systems, and challenges in providing services and strategies used to address them. These topics correspond to the questions asked to gynecologic cancer patients (Table 1). Additional details about our methodology are available elsewhere, including a report on environmental stressors experienced by gynecologic cancer patients and oncology care providers and administrators.^24^ The Institutional Review Board of the University of Puerto Rico Medical Sciences Campus approved the study protocol (#A1810418 approved on August 30, 2018), including the qualitative work involving both gynecologic cancer patients and oncology care providers and administrators.

### Qualitative Data Analysis

A bilingual professional transcriptionist transcribed focus group discussions and interviews that were entered into Atlas.ti for analysis. All study team members received 8 hours of training on qualitative data analyses and coding using Atlas.ti software before analyses. Six coders worked in pairs to read and code transcripts using a constant comparison method, consistent with the grounded theory approach.^25^ Coders used a thematic codebook for their coding assignments, which PML and MR developed with input from EAP, a qualitative research expert. The codebook was tested with an initial set of transcripts and refined with emerging topics. Transcripts were coded in Atlas.ti by reading the data line-by-line to identify concepts within each statement. Each data section was labeled according to the concept(s) in the transcript with a brief code and then used to create a new tree node. Repetitions of the same idea were coded to the same node, creating a list of recurring ideas. As coding developed and themes emerged, nodes were arranged in groups under a parent node labeled with the theme. The data classification and coding process under-identified themes involved building both categories (parent) and subcategories, which expanded as the review focus groups progressed. Beyond this stage, themes were collated into broader groups. An open coding was used when emerging topics were identified. We employed established methods for solving differences in coding themes by reconciling such discrepancies through group discussions and consensus.^25^ While we did not quantify the codes as this may provide skewed data (eg, one woman may mention the same theme many times), we indicated the potential frequency of themes using qualitative descriptors (eg, majority, several, few) when refering to all participants. The present qualitative work is part of a larger mixed methods study assessing the impact of hurricane-related stressors and responses on oncology care and health outcomes of women with gynecologic cancers.

## Results

Patients’ mean age was 57.5 years (standard deviation, 13.6 years; Table 2). Most patients had completed high school or higher education (77%), and almost half of them (42%) reported an annual household income of $15,000 or lower. The sample was comprised of women with diagnoses of ovarian (54%), corpus uteri (23%), and cervical (15%) cancers. Key informants were oncology care providers (eg, oncologists, radiotherapists), a pharmacist, hospital administrators, and representatives from government agencies and nonprofit organizations. Analyses of focus group discussions and interviews allowed us to identify 4 emergent themes: disruptions in oncology care, challenges in communication between oncology providers and patients, patient resilience, and must-needed social support and resources.

**Table 2.** Characteristics of Study Participants.

	N (%)
***Gynecologic cancer patients***	***26 (100)***
Residence	
Puerto Rico	24 (92)
U.S. Virgin Islands	2 (8)
Mean age, years (sd)	57.5 (13.6)
Education	
High school or less	6 (23)
Higher than high school	20 (77)
Marital status	
Divorced, separated or widowed	10 (38)
Married or live with partner	9 (35)
Single	5 (19)
Missing	2 (8)
Household income	
≤$15,000	11 (42)
>$15,000	15 (58)
Health insurance	
Private	16 (62)
Public	10 (38)
Gynecologic cancer	
Ovarian	14 (54)
Corpus uteri	6 (23)
Cervical	4 (15)
Missing	2 (8)
	**N (%)**
***Oncology care *** ***providers & administrators***	***23 (100)***
Sex	
Female	12 (52)
Male	11 (48)
Role	
Oncologic care	13 (57)
Pharmacist	1 (4)
Government personnel	4 (17)
Hospital administrator	2 (9)
Nonprofit personnel	3 (13)

### Theme 1: Disruptions in Oncology Care Were Common

The majority of participants expressed that the continuation of oncology care was a common problem in the wake of the hurricanes. Patients identified 2 major factors that caused substantial disruptions to the timely provision of care: the severely damaged health care system infrastructure and the lack of basic services like electricity and potable water. The damage to infrastructure was expressed in various ways, mostly the ruin of clinical facilities and flooding. Across the various regions in PR where focus groups took part, women noted that many buildings housing outpatient clinics, radiation oncology units, or pathology laboratories were affected by hurricane winds. Also, frequent damage to infrastructure was related to flooding. One patient mentioned, *“My doctor’s office was flooded, quite high in inches of water, everything was damaged. The patient records were damaged, and it was a disaster… [Patients] could not access that office for months.”* The second major issue that caused disruptions in care was the loss of electrical power and water in medical facilities. Several patients noted that even though some oncology care facilities were physically safe and had functioning backup generators, not all clinical areas were operating due to limited diesel supply and storage capacity. This observation was confirmed by many providers and administrators who noted the challenges experienced by their facilities in getting enough diesel to operate generators. Patients also noted that many medical facilities did not have power generators and simply did not open until the island’s electrical grid was restored. As one participant mentioned, *“I despaired of the fact that I was going [to clinics]… and they cannot do certain medical exams. There was no electricity in the clinics where I went, that was exasperating me.”* One provider told us that radiotherapy equipment requires a specific room temperature to be able to function, and until they did not have electricity to turn the clinic’s air conditioning at its normal capacity they could not deliver radiotherapy. Both damages to infrastructure and loss of basic services caused many delays in oncology care that women in our study experienced for up to 9 months. One patient said, *“I came every week in October and November after the hurricane, and they still didn’t give me treatment. The doctor told me that he was going to take me [to another hospital] and he was going to do the surgery there. I had surgery in June 2018.”* One participant from the USVI narrated, *“After surgery, I had to come to Puerto Rico every 4 months to visit the clinic for treatment. My surgery was in January 2017; it was before the hurricane. I was supposed to come back in October 2017, but I came in December. There were many problems due to the hit of the hurricane.”* Interviews with providers also showed that the impact of hurricanes on the timely continuation of oncology care was significant, with many clinics facing service interruptions of 2-4 weeks (Table 3).

**Table 3.** Example Quotes from Oncology Care Providers and Administrators.

Theme	Example Quotes
Disruptions in oncology care were common	“Most of the centers [radiotherapy facilities] in Puerto Rico had interruptions of 2 weeks, and some centers had interruptions of more than 1 month in treating radiotherapy patients.” “The greatest stressor [for patients] was the transportation, for example, patients who were in radiotherapy. Radiotherapy is daily and it was almost impossible if they did not have gasoline, if they did not have transportation to get to their radiotherapy center on a daily basis.” “Patients scheduled for surgery, we had to cancel the surgery for a few weeks, and not only surgeries but also post-operative services. So there were certain patients who decided not to have surgery, or if they had recently operated and wanted to continue their services in the USA, we helped them [to continue treatment outside of PR].”
Communication between oncology providers and patients was challenging before and after the hurricanes hit	“There were clinics that had the problem that they could not even call their patients because they could not access the electronic medical records to get the patients' phone numbers.” “The doctor did a lot of communications on the radio, Facebook live, television, she used all social networks to facilitate services and let people know that we were here [treating patients].” “The lack of communications, when it most affected us was when we received patient transfers without prior notice.” “We got an emergency telephone number, because our main telephone line was not working…so that those who could not reach our clinic, could call that number.”
Patient resilience was key to resume care	“Some patients who could not communicate, showed up in person, patients came [to the hospital] from different regions of the Island.” “Many [patients] went to emergency rooms looking for help because they did not know where to locate them [their oncology providers] because their doctor was no longer there [clinics were closed].”
Local communities provided much-needed social support and resources	“We distributed over one thousand bags with supplies. They [patients] came to get their treatment and left with a bag with toilet paper, razors, sanitary products, grocery…” “I know that a radiotherapy center in Mayagüez helped patients with transportation...”

### Theme 2: Communication Between Oncology Providers and Patients Was Challenging Before and After the Hurricanes Hit

The majority of patients expressed that, before Hurricane Maria hit, their oncology care providers had no plan in place to communicate with them to ensure continuity of care. In advance of the anticipated onset of Hurricane Maria, one woman remembered being contacted by her provider and receiving vague information, *“When they canceled my radiotherapy, they only told me that if I was feeling sick, that I could go to the emergency room for anything.”* During the hurricane warning period, another patient recalled, *“I did not receive any information on how I was going to continue with my treatment or a contingency plan from my doctor’s office.”* No patient from PR in our study mentioned receiving a copy of their last history of exams, regimen order summary, or lab results. In the aftermath, contacting clinics proved to be mostly useless because most telecommunication systems in the island were down. All patients agreed that cell phone service was spotty at best. Many patients remembered walking or driving to the few communication antennae towers left standing or climbing on their roofs to call their oncology clinics. However, they could not do so; one woman said, *“The cell phone signal strength was horrible, and I had to climb on the roof of my house to get a little bit of signal to call the clinic. As soon as it got some signal, I lost it.”* Providers and administrators also mentioned that the island’s telecommunication systems were down for many weeks but they tried multiple mechanisms to communicate with their patients including radio and television interviews, social media, and getting satellite-operated emergency telephones (Table 3).

### Theme 3: Patient Resilience Was Key to Resume Care

Most patients expressed that they felt relatively well prepared for hurricanes Irma and Maria because, as one participant expressed, *“we get hurricane and tropical storm warnings every year.”* Many patients also said they protected their homes accordingly and still had supplies they bought in preparation for Hurricane Irma, including canned food, potable water, batteries, and fuel for power generators. Few women said they were not prepared, blaming the lack of time or competing demands like job or family responsibilities. However, all patients agreed they never thought that the island would be hit so hard and that it would take them so long to get back to normal. One woman expressed that *“things are now more or less back to normal,”* and another said that *“things will never be the same.”* Despite the daily struggles, patients were constantly thinking about when to resume their oncology care. With communications down, several patients ventured to clinics with the expectation of finding them open. They mentioned listening through the radio or by word of mouth that some medical facilities throughout the island were resuming operations, but they did not know about their oncology clinics because of the lack of telecommunications. Some patients remembered driving for more than an hour, when it was usually a 20-minute drive, to reach the clinics because of the roads, and when they arrived, the clinic was closed. Others had better luck and found the clinics open; one woman said, *“I heard on the radio that the clinic was open, and I went there. [The clinic] did not have electricity yet but the doctor was seeing patients. However, the pathologist had not yet arrived to work so I had to wait again… The pathologist treated me in December.”* Regardless of the trip’s outcome, they needed to drive home while there was sunlight and before the law-enforced curfew started. One patient mentioned, *“I went to the doctor’s office [without appointment] because there was no telephone communication, I had to go straight to the medical offices… In the midst of all that trouble, leaving the house was a challenge because there was no electricity and the traffic lights were damaged. In addition, the clinics were opened on a reduced schedule, and there was a curfew in place.”* Providers also told us that some patients showed up in person to see whether clinics were resuming patient treatment services (Table 3).

### Theme 4: Local Communities Provided Much-Needed Social Support and Resources

Participants remembered that *“Hurricane Maria left the island in a state of chaos.” Patients* expressed high anxiety levels because they spent many days or weeks not knowing about their loved ones. Several patients also expressed anxiety because they were taking care of family members. One woman remembered, *“After the hurricane passed, the winds ended, not a single tree leaf moved… Oh my God! My mom was bathed in sweat every night, and you know, there was no water either. I cried, and I wondered how much more time we were not going to have electricity; it was very frustrating. I spent my nights watching over her because my fear was that because of so much heat, her sugar levels would drop and she would die sleeping.”* However, patients pleasantly recalled the continuous support they received from neighbors and community leaders, including getting hot meals, fuel for generators, and even help with some home repairs. As 1 woman expressed, *“Puerto Rican people are resilient.”* Many women agreed that community-level actions were fundamental in restoring access to neighborhoods and, ultimately, saving lives. Some providers and administrators mentioned that clinics helped patients by providing household supplies and transportation (Table 3). The majority of patients, providers, and administrators also agreed that, after the impact of Hurricane Maria, it took too much time for state and federal authorities to reach communities, especially those in rural areas. Some patients experienced significant losses of their homes and belongings. Fortunately, study participants reported that they and their household members were unharmed, but they recalled neighbors who died in the aftermath of the hurricanes. For all patients, the drastic lifestyle changes that followed the hurricanes constituted a dramatic departure from normality and resulted in an increase in stressors. These challenges greatly impacted the mental health of patients. One woman said, *“I was worried all the time; it was very frustrating for me, psychologically it affected me a lot.”* In these difficult times, however, patients witnessed solidarity and commitment to the restoration of communities. As a woman said, *“We even went to cook at the community center. We were supportive of the community. I say that despite the hurricane situation, serving others made me feel that I was returning goodness.”*

## Discussion

Hurricanes Irma and Maria posed extraordinary challenges for everyone in PR and the USVI, including immediate health dangers.^8^ But the literature shows that cancer patients are at more significant health risks than the general public because it might be difficult to get on-time oncology care, worsening their prognosis and survival.^16-18^ Women with gynecologic cancers included in this study revealed that disruptions in oncology care were common. Our findings are consistent with a recent study conducted in PR with 41 matched cancer survivors and non-cancer patient pairs.^26^ That study found that cancer survivors experienced more barriers to access medical care compared to non-cancer patients, especially in the first 6 weeks after Hurricane Maria.^26^ Patients in our study said that disruptions in cancer care were mostly caused by damage to health care infrastructure and loss of utility services. Their experiences align with findings from a recent systematic review showing that electricity and water supply are often damaged when hurricanes hit, leading to service disruption in cancer care facilities, resulting in extended closure of units and subsequent oncology treatment delays.^16^ Radiotherapy is particularly vulnerable because it requires dependable electrical power and the daily presence of highly-trained clinical staff for treatment delivery.^20^ Similar to the stories narrated by our study participants, the literature confirms that frequent damage to cancer care infrastructure following a hurricane includes flooding, which can cause costly damage to specialized radiotherapy equipment and clinical areas.^16^

Communication issues between oncology providers and patients were also a significant challenge experienced by study participants. Prior to hurricanes Irma and Maria, no patient recalled receiving a written or formal communication plan from their cancer care teams. It is well documented that, in the aftermath of hurricanes Irma and Maria, the prolonged loss of electricity and internet limited the ability of providers to communicate with their patients for weeks and even months.^4,8^ Emergency preparedness tips for cancer patients include discussing with the care team the possibility of getting an extra supply of medicines and who to call or where to go if patients cannot get through to them using regular telecommunication methods. The National Cancer Institute’s (NCI) Cancer Information Service (CIS) can help guide cancer patients to where to continue treatment if a disaster disrupts care, including communications with providers, or displaces them to other locales. The CIS can refer patients to nearby cancer treatment facilities or NCI Community Oncology Research Program (NCORP) sites. Similarly, organizations like the American Society of Clinical Oncology have called for improved regional oncology cooperative networks to better connect displaced patients, their oncologists, and those providers who are still providing care in the wake of disasters.^27^

Continuation and proper delivery of cancer care are among the top medical management priorities after a disaster.^16^ According to the United Nations’ Sendai Framework for Disaster Reduction: 2015-2030,^28^ “People with life-threatening and chronic disease, due to their particular needs, should be included in the design of policies and plans to manage their risks before, during, and after disasters, including having access to life-saving services.” While such plans should be housed within the Emergency Support Function (ESF) infrastructure of each U.S. jurisdiction (ie, federal authority to provide essential public health and medical services for disaster preparedness, response, and recovery),^29^ the Centers for Disease Control and Prevention (CDC)-sponsored cancer control plans provide an excellent community-centered mechanism to support these efforts.^30^ The CDC provides funds to all U.S. jurisdictions, including PR, to establish region-specific cancer control plans. Currently, these plans incorporate policy, systems, and built environment change interventions to support cancer control initiatives, so regional disaster plans would align well with the structure of local cancer plans.^30^ Equally important, a prior study from our team also identified that, following Hurricane Maria, environmental stressors such as lack of potable water, heat and uncomfortable temperatures, poor air quality, noise pollution from generators, and pests (eg, mosquitos, rats) were top concerns in PR.^24^ Future disaster plans should include strategies to mitigate these environmental stressors as they threaten public health and the well-being of populations.

Our interviews also showed that patients experienced emotional distress, especially those taking care of family members. At the same time, these women were resilient to resume their treatment and provide needed care to their families in the face of all adversity. Our observations are consistent with a biopsychosocial study reporting that Puerto Rican cancer survivors had greater levels of depression and greater resiliency vs non-cancer individuals in the aftermath of Hurricane Maria.^26^ The authors concluded that patients had a tendency to conceal emotional distress to protect loved ones from worrying.^26^ Our observations align with common cultural values in the Puerto Rican community and Hispanics in general, where family relationships are often prioritized before an individual’s well-being.^26,31^ Similar to other studies exploring people’s experiences post Hurricane Maria,^31^ we also found that local communities, including neighbors, local organizations, and clinics, provided support and resources to help patients with their financial, physical, and psychological needs.

### Strength and Limitations

A strength of our study was the diversity of participants, including the recruitment of women from 3 regions of PR and the USVI, the mix of privately and publicly insured patients, and the inclusion of oncology providers and administrators through key-informant interviews. Study limitations include the time between the hurricanes and data collection. Focus groups and interviews were conducted 15-18 months after the events, and participants might have experienced some recall bias. However, as noted by our participants, Hurricanes Irma and Maria shaped the lives of Puerto Ricans in such a way that they will never forget these events, even *“the sound made by the strong winds”* (quote from patient). Also, our interview guide was not designed to elicit diverse experiences in cancer care continuation by place of residence (ie, urban, suburban, or rural) among patients. Another study limitation was the small sample size, but the objective of this qualitative work was to elicit participants’ lived experiences with oncology care and not generalize the study findings. Studies are needed to quantitatively evaluate the impact of hurricane stressors and responses on oncology care and health outcomes, including accounting for differences by cancer type, time from diagnosis, type of treatment, and stage in therapeutic regimen. That is the focus of a retrospective cohort study being conducted by the authors, using a combination of medical chart review and survey data, with 400 women diagnosed with a gynocologic cancer between September 2016 and September 2018 (+/−1 year of the hurricanes).

### Conclusion

This qualitative work provided firsthand information about the disruptions in oncology care experienced by and the resiliency of women with gynecologic cancer following hurricanes Irma and Maria. In participants' own words, the disruptions in care resulted from damages suffered by health systems, lack of basic services island-wide, and the loss of communication with their oncology care teams. Patients also highlighted their challenges in taking care of family members and the role of communities in the coping mechanisms and disaster response to overcome physical and emotional needs. Our findings underscore the need to incorporate oncology care in the preparedness and response plans of communities, health systems, and local government agencies to maintain adequate care for cancer patients during and after disasters like hurricanes.
